# Effectiveness of face-to-face, blended and e-learning in teaching the application of local anaesthesia: a randomised study

**DOI:** 10.1186/s12909-021-02569-z

**Published:** 2021-02-27

**Authors:** Anna Bock, Kristian Kniha, Evgeny Goloborodko, Martin Lemos, Anne Barbara Rittich, Stephan Christian Möhlhenrich, Nicole Rafai, Frank Hölzle, Ali Modabber

**Affiliations:** 1 Department of Oral and Maxillofacial Surgery, University Hospital RWTH Aachen, Pauwelsstrasse 30, D-52074 Aachen, Germany; 2 Audiovisual Media Center, Medical Faculty, RWTH Aachen University, Pauwelsstraße 30, D-52074 Aachen, Germany; 3 Department of Prosthodontics and Biomaterials, University Hospital RWTH Aachen, Pauwelsstrasse 30, D-52074 Aachen, Germany; 4 Department of Orthodontics, University Witten/Herdecke, Alfred-Herrhausen-Straße 45, D-58448 Witten, Germany

**Keywords:** E-learning, Blended learning, Face-to-face learning, Local anaesthesia, Dental education

## Abstract

**Background:**

Local anaesthesia plays a key role in many aspects of a dentist’s work. The required skills to perform anaesthesia successfully are acquired at university. To take advantage of the possibilities for new teaching formats, a blended learning concept for the local anaesthesia course was developed. The aim of the study was to compare the effectiveness of face-to-face, blended and e-learning in teaching in local anaesthesia by assessing students’ knowledge gain, performance of practical skills and satisfaction with the course.

**Methods:**

All participants (*n* = 37) were randomly allocated into three groups. After acquiring the theoretical background in the blended learning, e-learning or lecture groups, a test to assess knowledge gain was performed. The performance of the practical skills was assessed in a small-group seminar. Student attitudes were evaluated with a questionnaire.

**Results:**

The blended group showed significantly better results (mean = 17, SD =1.5) in theoretical knowledge gain than the other two groups (e-learning group: mean = 14.7, SD = 2.2; lecture group: mean = 14.8, SD =2.3). When comparing the results of the clinical skills assessment, there was no significant difference among all three groups (*p* > 0.017). The participants confirmed a high overall satisfaction with the course, in particular with the blended learning approach.

**Conclusion:**

This study indicates that blended learning improves the learning outcome for theoretical knowledge in teaching local anaesthesia more than either face-to-face learning or e-learning alone. Furthermore, the blended learning approach is highly appreciated by the students. For acquiring practical skills, this study shows that blended learning is as effective as other teaching methods.

**Supplementary Information:**

The online version contains supplementary material available at 10.1186/s12909-021-02569-z.

## Background

Local anaesthesia is used to induce insensitivity to pain in a defined small part of the body. In dental practice, it plays a key role in completing several dental procedures, for example periodontal treatment, endodontic treatment and oral surgery. Therefore, dentists administer local anaesthesia several times a day in order to reduce patients’ distress and pain [1]. To perform local anaesthesia successfully, the clinician needs to have a precise knowledge of anatomy, indications and complications [2].

In particular, block anaesthesia of the inferior alveolar nerve can be challenging because of anatomical variations. The literature describes that the success rate of conventional inferior alveolar nerve block is only 80–85% [3]. Since it is so important in the daily clinical life of a dentist, sufficient and effective instruction is needed when teaching dental students.

Dentists generally gain knowledge of local anaesthesia and practical skills at university. Besides the different types of local anaesthesia, application, side effects and complications are discussed in class. Apart from the theoretical knowledge about local anaesthesia, the practical implementation needs to be acquired before graduation. The acquisition takes place during the dental treatment in the courses under supervision of the course leaders. According to the new developments of teaching methods in medical and dental education, blended learning, the combination of digital media and traditional classroom teaching, is a promising approach to transforming traditional lectures into modern and sustainable teaching formats. Applying blended learning to a course results in a technology-enhanced learning experience [3]. Several studies have shown that dental students receive blended learning positively and have a high satisfaction using it. For example, Bock et al. integrated the blended learning approach to a traditional lecture in order to transform it into a modern, sustainable, and technology-enhanced learning experience, which was appreciated by the students of dental education [4]. Varthis et al. used the blended learning approach in a flipped classroom format for teaching theoretical knowledge about maxillofacial prosthetics. Students participating in this study reported positive perceptions of the blended learning [5]. Moreover, when assessing knowledge gain, a study by Munro et al. has shown that blended learning is at least as effective, if not more so, as traditional teaching formats when assessing self-management skills in physiotherapy [6]. So far, the positive results of blended learning are mostly for acquiring theoretical knowledge and not for acquiring practical skills. There are only a few studies reporting that blended learning may be as effective as face-to-face learning in improving practical skills in medical eduaction [7–10]. For example, Perkins et al. compared conventional advanced life support training with a blended learning approach and showed that it led to similar scores on a knowledge test and comparable results on the skills test [7]. In dental education, the effectiveness of blended learning approaches for training practical skills has not been described.

To address this lack of research, the aim of the study was to compare the effectiveness of face-to-face, blended learning and e-learning in teaching local anaesthesia by assessing students’ knowledge gain, performance of practical skills and satisfaction with the course.

## Methods

### General course design

At our university, anaesthetic injections are taught by the Department for Oral and Maxillofacial Surgery for dental students in the 3rd year before the treatment of real patients is started. The course consists of two parts. The first part is a traditional face-to-face lecture about the theoretical background of local anaesthetic injections. The second part is a practical seminar in small groups. At the beginning of the seminar, an infiltration anaesthesia and a block anaesthesia at the inferior alveolar nerve and the infraorbital nerve are demonstrated by a lecturer. Then the students practise the local anaesthesia on each other under supervision. After this course, the newly gained knowledge is applied and practised during the dental treatment in following student courses until graduation.

### Study design

In the summer term 2019, dental students in the 6th semester were invited to participate in the study. All registered students voluntarily agreed to participate at the study. Informed consent was obtained. All methods were carried out in accordance with relevant guidelines and regulations.

Before the course, all participants (*n* = 37) were allocated into three groups by simple randomization alphabetically. The first group, the lecture group (*n* = 12), attended the regular lecture. At the same time as the lecture, the second group, the e-learning group (n = 12), received independent study time with an e-learning programme in a computer room. The duration of the lecture and the independent study time was 1.5 h. The lecture itself was passive although questioning was allowed. There were no formative questions in the lecture. The content of the lecture and the e-learning programme was identical. The third group, the blended group (*n* = 13), were supposed to study using the e-learning programme before the lecture. The blended group and lecture group attended the same lecture at the same time, delivered by the same lecturer. The blended group received their login details for the e-learning programme 5 days before the lecture and were free to use it as intensively as desired.

Immediately after the lecture and the independent study time, a test consisting of 20 multiple-choice questions with one correct option out of five was administered. The maximum score of the test was 20 points. There were no partial or subtracted points. This theory test assessed the content of the e-learning programme and lecture. All questions were approved by the course leaders.

**Fig. 1:**
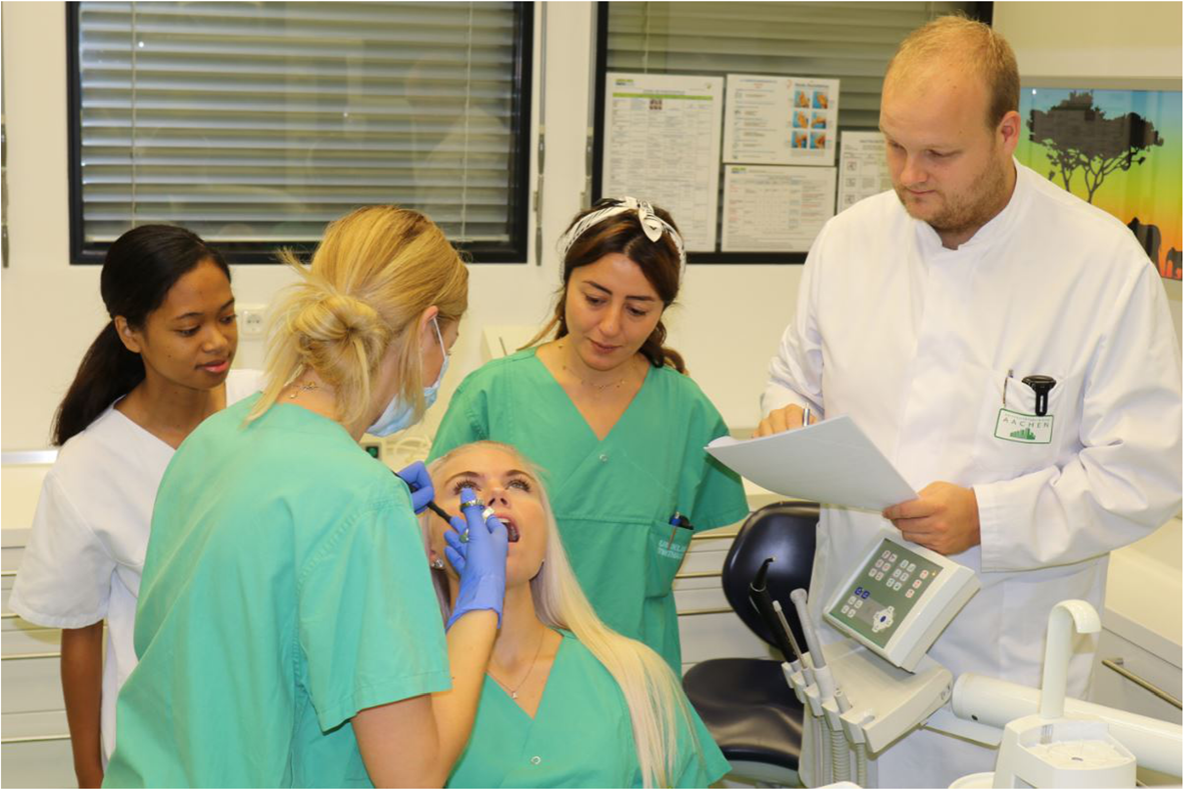
Setting of a practical seminar. The injection is performed under supervision of the lecturer

One week later, a practical seminar on how to perform injections of local anaesthesia took place. All seminars were groups of six participants (separated by group) and supervised by the same lecturer (Fig. 1). The lecturer was blinded to which of the three groups the students were in. After a short introduction and the demonstration of a block anaesthesia, each participant had to perform a block anaesthesia on another participant, while the other students were able to observe. This performance was evaluated by the lecturer on an assessment form composed in advance (see supplementary material). Each act was rated on a 9-point scale, with 1 indicating “insecure” and 9 “secure”. The maximum score for the assessment was 180 points. The feedback on the performance was provided at the end of seminar, after all participants were assessed. At the end of the seminar, the course was evaluated anonymously. The evaluation questions were answered on a ten-point Likert-scale, 1 indicating “fully agree/unsatisfactory” and 10 “totally disagree/satisfactory.” The survey consisted of 3 sections, demographic information, e-learning and the seminar/course. (see supplementary material).

### E-learning

**Fig. 2:**
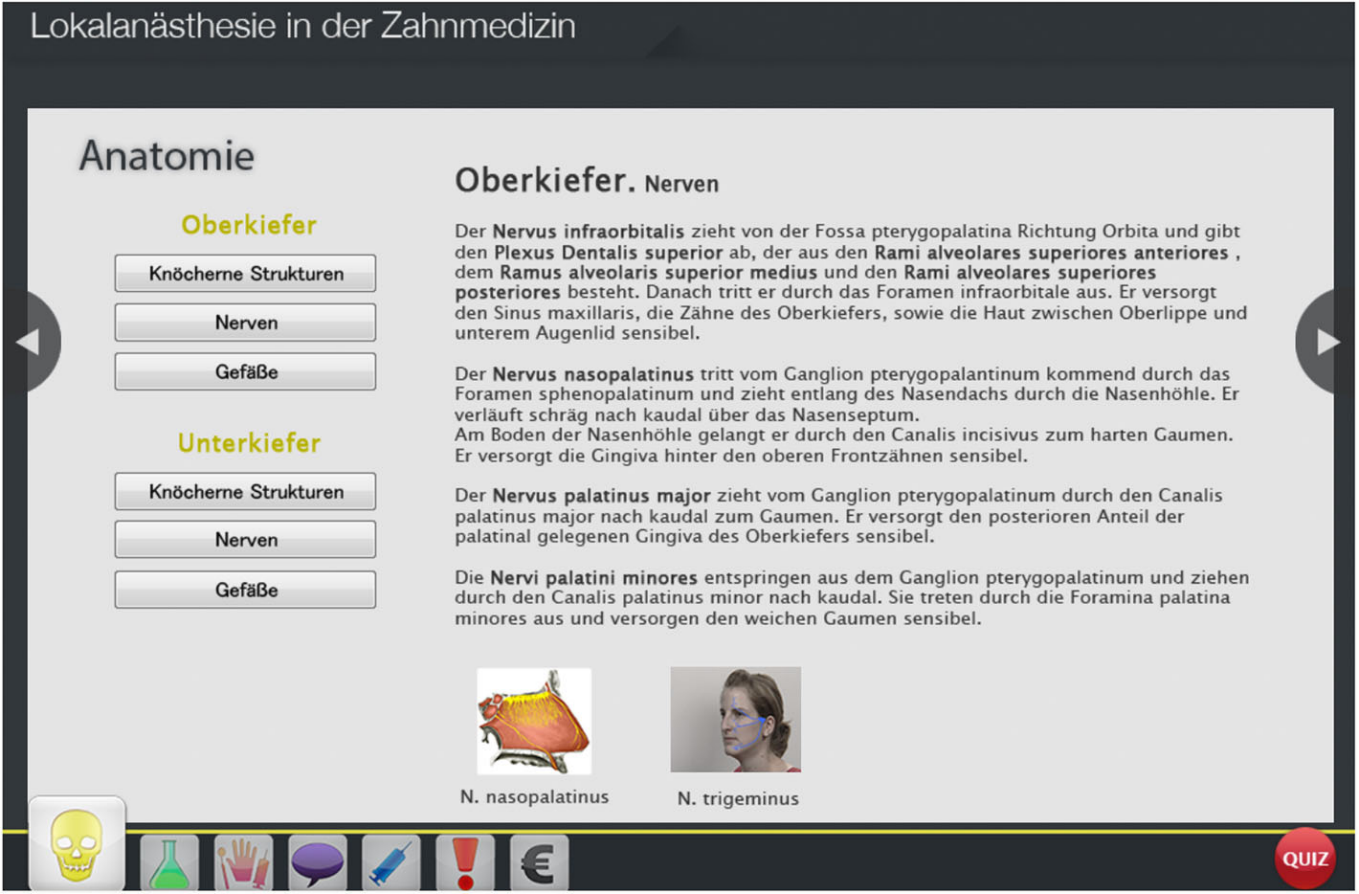
A screenshot of the e-learning programme showing the first section ‘anatomy’. On the left side, the section is subdivided into the anatomy of the upper and the lower jaw, displaying and explaining the bony structures, nerves and vessels of each region

The e-learning programme about local anaesthesia consists of seven chapters. The first chapter serves to refresh the anatomy of the upper and lower jaw, including bony structures, nerves and blood vessels, as displayed in Fig. 2. The second chapter deals with the pharmacokinetics of local anaesthesia. The third chapter explains the different materials being used when performing local anaesthesia. This chapter consists of a written portion and a video demonstrating the handling of the materials. The fourth chapter points out the obligations of patient information, documentation and consequences. The fifth chapter consists of seven videos demonstrating local injection at different intraoral anatomical sites. The sixth chapter explains the possible complications when using a local anaesthesia, including intoxication, nerve injury, allergic reactions and vasovagal syncope. The last chapter deals with the German accounting system, which is used in daily clinical life. Additionally, the e-learning program contains a formative (practice) quiz with 8 questions.

### Statistics

The obtained data was arranged using MS Office Excel 2016 (Microsoft Corporation, Redmond, Washington, USA). Statistical analyses were conducted using GraphPad Prism 6 Software (GraphPad Software, San Diego, CA). An unpaired t-test was used to compare the results of the three groups of the theoretical test and the clinical skills assessment, after normal distribution was checked through a D’Agostino-Pearson normality test in omnibus K2 variant. To avoid Type I error in the pair-wise comparisons of groups, the Bonferroni correction was applied to the *P* value. *P* < 0.017 was considered statistically significant.

## Results

### Participants

In total 37 students participated in this study. There were no dropouts. Out of all participants, 54% were male and 46% were female. Twelve participants were < 22 years, 16 were between 22 and 25 years, and nine participants were older than 25 years old.

### Theoretical test

**Fig. 3:**
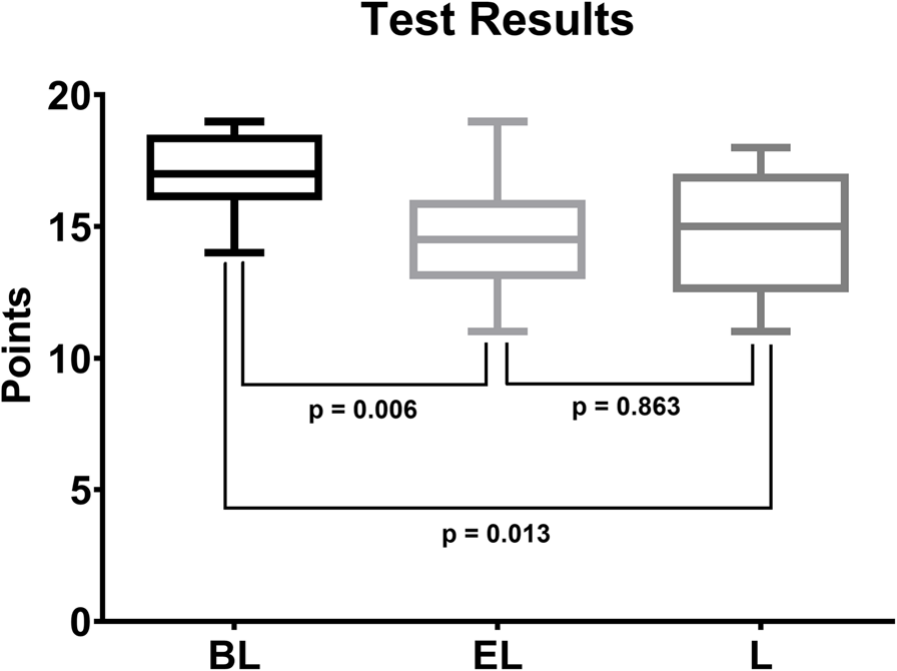
Boxplot displaying the results of the theoretical test. The blended group (BL) showed significantly better results than the e-learning (EL) and lecture (L) groups (*p* < 0.017)

The results of the theoretical test are displayed in Table 1 and Fig. 3. Comparing the test scores of all three groups, the blended group showed significantly better results than the two other groups (*p* = 0.013 and *p* = 0.006). There was no statistical significance between the results of the lecture group and the e-learning group (*p* = 0.863).

**Table 1:** Results of the theoretical test and the clinical skills assessment

	Theoretical Test		Clinical Skills	
	Mean test score	Standard deviation	Mean test score	Standard deviation
Lecture Group	14.8	2.3	161	12
E-learning Group	14.7	2.2	154	22
Blended Group	17	1.5	152	23

### Clinical skills

**Fig. 4:**
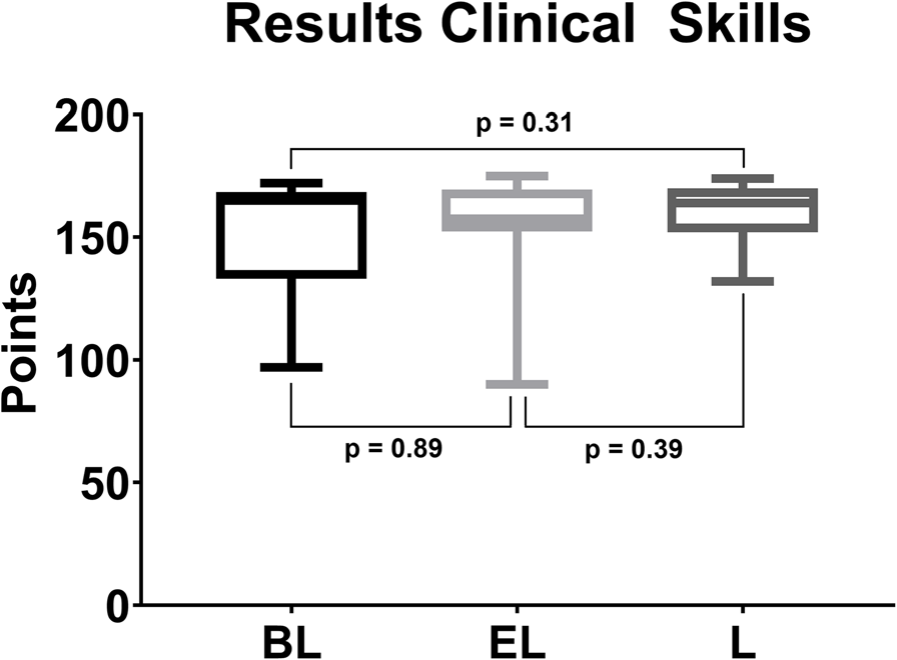
Boxplot displaying the results of the practical assessment. There were no significant differences amongst the blended (BL), e-learning (EL) and lecture (L) groups (*p* > 0.017)

The results of the clinical skills assessment are displayed in Table 1 and Fig. 4. There were no significant differences between all three groups (*p* > 0.017). Also, the results of the subitems preparation, skill in block anaesthesia of the inferior alveolar nerve and block anaesthesia of the infraorbital nerve did not show significant differences (*p* > 0.017).

### Evaluation

Participants of both groups using e-learning (*n* = 25) evaluated the e-learning programme. The results are shown in Table 2. There were no remarkable differences in the two groups’ ratings of the e-learning program. Both groups confirmed that the e-learning is a useful supplement to the course (mean score = 2.56, SD = 2.53). They did not think that e-learning could replace the practical seminars (mean score = 9.32, SD = 1.46). In their opinion, the lecture should also not be replaced by the e-learning programme (mean score = 6.56, SD = 2.14).

**Table 2:** Results of the evaluation of the e-learning programme. The questions were answered on a ten-point Likert-scale, 1 indicating “fully agree” and 10 “totally disagree”

Aspects	Mean score	Standard deviation
The layout of the programme has a clear presentation.	2.92	0.89
The programme has an intuitive interface.	2.68	1.41
The programme is didactically well designed.	2.4	1.06
The programme has a good amount of educational content.	2.76	1.56
The programme motivated me to learn more.	4.17	1.86

All students of the blended learning group confirmed that they used the e-learning programme to prepare for the course. They indicated their high satisfaction with the teaching approach (mean score = 1.62, SD = 0.49). The e-learning group (mean score = 1.67, SD = 0.47) and the lecture group (mean score = 1.58, SD = 0.64) expressed their high satisfaction with the course as well.

**Fig. 5:**
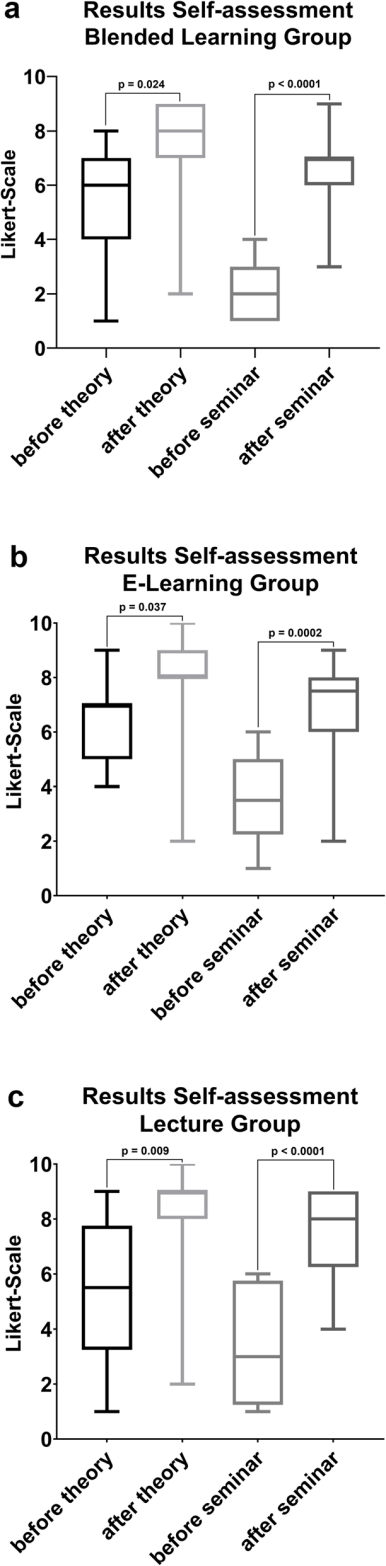
Boxplot displaying the self-assessment of all three groups for the theoretical and the practical part. The median of the blended group after seminar is 7.5. The medians of the e-learning group are before the theory ist 6 and after theory 8. The median of the lecture group after the theory is 9

Regarding the self-assessment, all three groups confirmed a theoretical knowledge gain and an improvement of their practical skills after the seminar, which was statistically significant (Fig. 5). There were no statistical differences in the self-assessments between the groups. After the practical seminar students of all groups felt fairly well prepared to perform the procedure on a patient (mean score = 3.97, SD = 2.03). They indicated that practising on each other was very useful (mean = 1.46, SD = 1.22).

## Discussion

This study intended to compare the effectiveness of face-to-face, blended and e-learning in teaching in local anaesthesia. Therefore, the students’ knowledge, performance of practical skills and satisfaction was assessed. The blended learning group showed significantly better results in the test assessing theoretical knowledge than the two other groups. This confirms the results of other studies, suggesting that blended learning is more likely than either face-to-face learning or e-learning alone, to be effective in knowledge acquisition [10, 11]. Additionally, these studies have shown a high acceptance of the blended learning approach by the students [10, 11]. Moreover, blended learning has been shown to generate high levels of student satisfaction [12–14]. Our evaluation shows similar results, with participants confirming a high level of satisfaction with the blended learning approach. Nevertheless, the e-learning group expressed high satisfaction with the e-learning in combination with the practical seminar to acquire the practical skills as well. A major advantage of e-learning is the possibility of self-directed learning. Self-directed learning is defined as an individual process whereby students take the initiative in determining their learning needs and goals [15]. Besides taking individual learning style and speed into account, e-learning generally is independent from time and place, although in this study the e-learning group had to work through the program at the same time the other two groups attended the lecture [16, 17]. Additionally, e-learning might offer easier access to information with wider variety and greater quantity [18, 19]. This enables optimised conditions for the learning process. Studies have shown that e-learning modalities are used widely by students outside of their formal curricula and by health professionals for continuing professional education, indicating that students and professionals appreciate and take advantage of e-learning [20–22]. For example, Rapp et al. found out that YouTube was the preferred internet source for surgical videos in order to prepare for surgery [21]. In the present study, the blended learning approach provided a ‘structured preparation’ for the lecture and practical seminar; this may be preferable to leaving students to prepare on their own for the course.

Regarding the results of the self-assessment of the theoretical knowledge gain, all three groups assessed that their knowledge gain was similar. Each group described a significant theoretical knowledge gain by attending the first part of the course, but there were no differences amongst the groups. This suggests that the lecture and the e-learning programme are appropriate to gain knowledge. Nevertheless, according to the theory test results, the blended learning approach appears to be superior to traditional face-to-face teaching or e-learning alone.

Lecturer assessment of practical skills revealed there was no significant difference observed in the performance of all three groups. These objective results correspond to the subjective results of the self-assessment of the practical skills. All three groups assessed their improvement of the performance as equally good. In our study, both groups using the e-learning programme had already watched the educational videos on how to perform local and block anaesthesia ahead of the seminar. Still, there was a demonstration by the lecturer for each group how to perform the procedure at the beginning of the practical seminar. The lecture group therefore had the chance to align their knowledge of the practical procedure. This reflects the results of other studies showing that for practical skills, blended learning is as effective as traditional teaching methods like face to face learning [7–10, 23]. Nevertheless, there are some other studies reporting that blended learning may significantly improve practical skills so that there are inconsistencies in the literature [24, 25]. A systematic review by George et al. rated the quality of evidence for all these studies low [26].

In the evaluation, students pointed out that the practising of local anaesthesia on each other under supervision and the observation of the other participants was very useful for their learning process.

Additionally, the evaluation revealed that the participants do not agree that the practical seminar can be replaced by e-learning alone. Rather, e-learning should be used as a complementary learning opportunity, as in blended learning approaches. This confirms the results of other studies pointing out that e-learning is accepted as supplementary learning option and should not replace personal interaction [27, 28]. Similarly, students are more satisfied with their learning when there is a perceived high level of social interaction and collaboration [29].

The study has some limitations. Apart from the small sample size, all groups have been instructed not to reveal their course materials to their peers of other groups. Still, the risk of cross-contamination amongst groups remains a possibility. A significant bias is that the blended group was offered more chances to gain theoretical knowledge, since they attended the lecture and had the chance to use the e-learning programme ahead as much as desired. Since no usage data was obtained and students were not asked in the evaluation, we do not know how much the e-learning programme was actually used before the lecture in the blended group. The other two groups only attended the lecture or used the e-learning programme. Nevertheless, it is possible that the participants of these two groups prepared independently for the course with books or the internet for example. Other studies, suffer from the same limitations, as blended groups were offered more time for learning [6, 30, 31]. Another limitation is that the groups using the e-learning programme were offered an 8-question formative (practice) test with feedback, so that they had the opportunity to rehearse their knowledge prior to the 20-question knowledge test. However, none of the formative questions were repeated on the knowledge test. Another important aspect is the possibility of a recall bias when asking the students to rate their knowledge and practical skills before and after the seminar after both had occurred. Generally it is better to rate the knowledge and practical skills before undertaking the seminar and again after the seminar. Furthermore, only short-term retention was assessed in our study. Due to organisational reasons, many studies assess only the short-term retention [6–9, 14, 15, 30, 31]. The assessment of long-term retention would be important to assess in future research.

## Conclusion

This study indicates that blended learning in teaching local anaesthesia improves the learning outcome for theoretical knowledge more than either face-to-face learning or e-learning alone. For acquiring practical skills, blended learning is as effective as other teaching methods. Nevertheless, the blended learning approach is highly appreciated by the students and should be applied to the course. Future studies should investigate possibilities for blended learning approaches to improve the development of practical skills.

## Supplementary Information

**Additional file 1.** Evaluation Form.

**Additional file 2.** Assessment Practical Skills.

## Data Availability

The datasets used and/or analysed during the current study are available from the corresponding author on reasonable request.
