# Supplementary material for: Effectiveness of face-to-face, blended and e-learning in teaching the application of local anaesthesia: a randomised study
Source: BMC Med Educ. 2021 Feb 27;21:137. doi: 10.1186/s12909-021-02569-z (PMC7913455; doi:10.1186/s12909-021-02569-z)
Supplement: Supplementary file 1 — Additional file 1. Evaluation Form. [file 12909_2021_2569_MOESM1_ESM.pdf]

Evaluation Form

<u>General informations</u>

1.1 Gender: □ F □ M

1.2 Age: □ <22 J. □ 22-25 J. □ >25 J.

1.3 Study year: □ 3rd year □ 4th year

1.4 Group: □ Lecture

□ E-learning

□ Blended learning

<u>E-learning</u>

2.1 The e-learning programme is an useful supplement to the course.

□1 □2 □3 □4 □5 □6 □7 □8 □9 □10

Totally agree Totally disagree

2.2 The e-learning programme could replace the practical seminar.

□1 □2 □3 □4 □5 □6 □7 □8 □9 □10

Totally agree Totally disagree

2.3 The e-learning programme could replace the lecture.

□1 □2 □3 □4 □5 □6 □7 □8 □9 □10

Totally agree Totally disagree

2.4 Did you use the e-learning programme for preparation of the course?

□ yes □ no

2.5 The layout of the programme has a clear presentation.

□1 □2 □3 □4 □5 □6 □7 □8 □9 □10

Totally agree Totally disagree

2.6 The programme has an intuitive interface.

□1 □2 □3 □4 □5 □6 □7 □8 □9 □10

Totally agree Totally disagree

2.7 The programme is didactically well designed.

□1 □2 □3 □4 □5 □6 □7 □8 □9 □10

Totally agree Totally disagree

2.8 The programme has a good amount of educational content.

□1 □2 □3 □4 □5 □6 □7 □8 □9 □10

Totally agree Totally disagree

2.9 The programme motivated me to learn more.

□1 □2 □3 □4 □5 □6 □7 □8 □9 □10

Totally agree Totally disagree

Effectivity of the course

3.1 Thanks to the seminar, I feel well prepared to perform the procedure on a patient.
□1 □2 □3 □4 □5 □6 □7 □8 □9 □10
Totally agree Totally disagree

3.2 My theoretical knowledge before the seminar was
□1 □2 □3 □4 □5 □6 □7 □8 □9 □10
Unsatisfactory Satisfactory

3.3 My theoretical knowledge after the seminar was
□1 □2 □3 □4 □5 □6 □7 □8 □9 □10
Unsatisfactory Satisfactory

3.4 I felt secure about performing local anaesthesia before the seminar?
□1 □2 □3 □4 □5 □6 □7 □8 □9 □10
Totally agree Totally disagree

3.5 I felt secure about performing local anaesthesia after the seminar?
□1 □2 □3 □4 □5 □6 □7 □8 □9 □10
Totally agree Totally disagree

3.6 Practising the performance of local anaesthesia on each other is very useful.
□1 □2 □3 □4 □5 □6 □7 □8 □9 □10
Totally agree Totally disagree

3.7 Overall satisfaction with the course and the teaching approach.
□1 □2 □3 □4 □5 □6 □7 □8 □9 □10
Unsatisfactory Satisfactory
