# Supplementary material for: Effectiveness of face-to-face, blended and e-learning in teaching the application of local anaesthesia: a randomised study
Source: BMC Med Educ. 2021 Feb 27;21:137. doi: 10.1186/s12909-021-02569-z (PMC7913455; doi:10.1186/s12909-021-02569-z)
Supplement: Supplementary file 2 — Additional file 2. Assessment Practical Skills. [file 12909_2021_2569_MOESM2_ESM.pdf]

Assessment form: **Local anaesthesia**
Department of Oral- and Maxillofacial Surgery

Prename: ____________________________

Name: ____________________________

Date:

Group: ☐ Lecture ☐ E-learning ☐ Blended Learning

	**Requirement**									
	**partly fulfilled/ insecure**			**fulfilled**			**outperformed/ experienced**			**Not applicable/ irrelevant**
	**1**	**2**	**3**	**4**	**5**	**6**	**7**	**8**	**9**	
**Preparations**										
• appropriate hand desinfection	☐	☐	☐	☐	☐	☐	☐	☐	☐	☐
• putting on gloves	☐	☐	☐	☐	☐	☐	☐	☐	☐	☐
• appropriate preparation of all required instruments (mirror, sucker, light)	☐	☐	☐	☐	☐	☐	☐	☐	☐	☐
• preparation local anaesthesia (refilling the syringe, venting)	☐	☐	☐	☐	☐	☐	☐	☐	☐	☐
**Implementation block anaesthesia of inferior alveolar nerve**										
• presentation of injection site trough holding and tightening the soft tissue	☐	☐	☐	☐	☐	☐	☐	☐	☐	☐
• correct selection of injection site (correct region)	☐	☐	☐	☐	☐	☐	☐	☐	☐	☐
• correct stance of needle and penetration of mucosa (needle comes from premolar region of the opposite site)	☐	☐	☐	☐	☐	☐	☐	☐	☐	☐
• protrusion of needle until bone contact	☐	☐	☐	☐	☐	☐	☐	☐	☐	☐
• aspiration	☐	☐	☐	☐	☐	☐	☐	☐	☐	☐
• slow injection	☐	☐	☐	☐	☐	☐	☐	☐	☐	☐
• appropriate removal and filing oft he needle	☐	☐	☐	☐	☐	☐	☐	☐	☐	☐
• patient observation awaiting the effect of the anaesthesia	☐	☐	☐	☐	☐	☐	☐	☐	☐	☐

**Implementation block anaesthesia of infraorbital nerve**										
• presentation of injection site trough holding and tightening the soft tissue	☐	☐	☐	☐	☐	☐	☐	☐	☐	☐
• correct selection of injection site (correct region)	☐	☐	☐	☐	☐	☐	☐	☐	☐	☐
• correct stance of needle (grinding facet of the needle turned down)	☐	☐	☐	☐	☐	☐	☐	☐	☐	☐
• aspiration	☐	☐	☐	☐	☐	☐	☐	☐	☐	☐
• slow injection	☐	☐	☐	☐	☐	☐	☐	☐	☐	☐
• appropriate removal and filing oft he needle	☐	☐	☐	☐	☐	☐	☐	☐	☐	☐
• patient observation awaiting the effect of the anaesthesia	☐	☐	☐	☐	☐	☐	☐	☐	☐	☐
**Disposal**										
• appropriate disposal of needle	☐	☐	☐	☐	☐	☐	☐	☐	☐	☐
